# Transcriptomic changes in liver transplant recipients with non-alcoholic steatohepatitis indicate dysregulation of wound healing

**DOI:** 10.3389/fendo.2023.1111614

**Published:** 2023-05-08

**Authors:** Diogo Pellegrina, Khairunnadiya Prayitno, Amirhossein Azhie, Elisa Pasini, Cristina Baciu, Sandra Fischer, Jüri Reimand, Mamatha Bhat

**Affiliations:** ^1^ Computational Biology Program, Ontario Institute for Cancer Research, Toronto, ON, Canada; ^2^ Multi-Organ Transplant Program, Toronto General Hospital Research Institute, University Health Network, Toronto, ON, Canada; ^3^ Department of Laboratory Medicine and Pathology, University of Toronto, Toronto, ON, Canada; ^4^ Department of Molecular Genetics, University of Toronto, Toronto, ON, Canada; ^5^ Department of Medical Biophysics, University of Toronto, Toronto, ON, Canada; ^6^ Ajmera Transplant Center, Toronto General Hospital, University Health Network, Toronto, ON, Canada; ^7^ Division of Gastroenterology & Hepatology, Department of Medicine, University of Toronto, Toronto, ON, Canada

**Keywords:** liver transplantation, non-alcoholic fatty liver disease, non-alcoholic steatohepatitis, disease pathogenesis, fibrosis, wound healing, transcriptomics (RNA-seq), computational biology & bioinformatics

## Abstract

**Background:**

Non-alcoholic steatohepatitis (NASH) has become a leading indication for liver transplantation. However, it often recurs in the graft and can also arise *de novo* in individuals transplanted for other indications. Post-transplant NASH (PT-NASH) is more aggressive and leads to accelerated fibrosis. The mechanistic basis of PT-NASH has not yet been defined and no specific therapeutic strategies are currently available.

**Methods:**

Here, we profiled the transcriptomes of livers with PT-NASH from liver transplant recipients to identify dysregulated genes, pathways, and molecular interaction networks.

**Results:**

Transcriptomic changes in the PI3K-Akt pathway were observed in association with metabolic alterations in PT-NASH. Other significant changes in gene expression were associated with DNA replication, cell cycle, extracellular matrix organization, and wound healing. A systematic comparison with non-transplant NASH (NT-NASH) liver transcriptomes indicated an increased activation of wound healing and angiogenesis pathways in the post-transplant condition.

**Conclusion:**

Beyond altered lipid metabolism, dysregulation of wound healing and tissue repair mechanisms may contribute to the accelerated development of fibrosis associated with PT-NASH. This presents an attractive therapeutic avenue to explore for PT-NASH to optimize the benefit and survival of the graft.

## 1 Introduction

Non-alcoholic fatty liver disease (NAFLD) is rising alongside the global epidemics of diabetes and obesity. One fourth of the global population and one third of North Americans are estimated to have NAFLD, encompassing a disease spectrum ranging from steatosis to non-alcoholic steatohepatitis (NASH) and cirrhosis (1, 2). Fat accumulation leads to inflammation, whereby wound healing is induced to repair tissue damage that, if left unchecked, can slowly develop into scarring and liver failure (3).

The increasing incidence of NASH has increased the necessity for liver transplantation (LT). In fact, NASH has become one of the top indications of LT globally (4). Accordingly, post-transplant NAFLD and NASH (PT-NASH) are common and associated with compromised survival after LT (2). While non-transplant NAFLD and NASH (NT-NASH) remain silent and progress at a rate of one stage per 14 years and seven years respectively (2), post-transplant diseases develop more rapidly, with almost half of recipients with NAFLD/NASH presenting recurrence with stage three fibrosis within five years (5).

PT-NASH is an aggressive disease that develops in a “normal” graft of LT recipients, but its molecular basis is not well understood. Previous studies have compared NT-NASH livers to healthy livers and found transcriptomic correlations with disease progression (6–8). Liver signatures of NT-NASH are largely overlapping with NAFLD but distinct from healthy livers. These signatures have been related to the dysregulation of lipid metabolism, immunomodulation, extracellular matrix remodeling, and cell cycle control (6). Here, we highlight transcriptomic changes that occur in human PT-NASH.

## 2 Materials and methods

### 2.1 Patient cohort

Adult LT recipients with post-transplant biopsies performed at University Health Network (UHN) (Toronto, ON, Canada) during 2016-2020 were selected by a pathologist and classified using the NAFLD activity scoring (NAS) (9) and METAVIR scoring for fibrosis (10). Archived fresh-frozen liver biopsy samples were collected from the Multi-Organ Transplant Biobank, including four samples with evidence of PT-NASH (two recurrent and two *de novo*), two with PT-steatosis (both *de novo*), and four controls with normal liver histology. This protocol was approved by the UHN Institutional Research Ethics Board (REB #17-5102).

### 2.2 RNA preparation and sequencing

RNA was isolated from fresh-frozen tissue using the RNeasy Mini Kit (Qiagen) and evaluated on a Bioanalyzer (Agilent). Libraries were prepared using the KAPA RNA HyperPrep Kit with RiboErase (Roche) and sequenced on Illumina NovaSeq 6000 at the genomics core of Ontario Institute for Cancer Research (OICR). Sequencing runs were performed at 2x151 bps to the depth of 100 million paired end reads. Raw data are available in ArrayExpress (accession E-MTAB-11688).

### 2.3 Data alignment

Unstranded paired reads with inward orientation were pseudo-aligned using Salmon method (version 1.5.1) (11) and the human genome GRCh38.p13 (Ensembl) with default parameters. Genes were quantified as transcript per million (TPM) values.

### 2.4 Differential expression analysis

The DESeq2 R package (12) with tximport was used for differential expression analysis using a binary design comparing PT-NASH samples with controls. Differentially expressed genes (DEGs) were filtered for false discovery rate (FDR) ≤ 0.05. Genes with small fold changes (|log2 FC| ≤ 1), low expression (mean ≤ 0.1 TPM) and/or missing P-values were removed. To study changes in disease progression, we defined the genes as monotonic or non-monotonic, by comparing average TPM values in control, PT-steatosis, and PT-NASH samples. Genes classified as monotonic showed increasing or decreasing TPM changes in disease progression while non-monotonic genes showed extreme values in the PT-steatosis group. Pearson correlation distance was used for clustering.

### 2.5 Re-analysis of RNA-seq data of non-transplant NASH

To compare our data with NT-NASH transcriptomes, we reanalyzed a previously published RNA-seq dataset of human livers with NAFL (steatosis), NT-NASH, and healthy controls (GSE126848) (6). We performed the DESeq2 differential gene expression analysis using the read counts dataset of the original study and converted these to TPM values using Ensembl GRCh38 for transcript lengths. Significant DEGs were identified using the following filters: FDR ≤ 0.01, |log2 FC| ≥ 1, TPM ≥ 0.1. Due to the increased size and statistical power of this dataset, we performed down-sampling and DESeq2 analysis of 1000 sets of four control and four NT-NASH samples, and conservatively assigned each gene the median down-sampled P-value followed by FDR correction.

### 2.6 Functional enrichment analysis

Pathway enrichment analysis was performed using the ActivePathways method (version 1.0.2) (13) and its default parameters. Gene sets of Gene Ontology (GO), wikipathways, and REACTOME were used for functional annotations (retrieved from the g:Profiler website, 2021-11-07). Significantly enriched pathways (FDR < 0.2) were visualized using EnrichmentMap and Cytoscape with standard protocols (14).

### 2.7 Protein-protein interaction networks

We constructed a protein-protein interaction (PPI) network of all DEGs found in PT-NASH and NT-NASH using BioGRID (15). The primary PPI network included the interactions of DEGs defined above. To study additional sub-significant genes, we included a secondary PPI network of interactions with less-stringent DEGs (FDR < 0.2).

## 3 Results

### 3.1 Clinical characteristics

We identified six available samples from LT recipients diagnosed with PT-steatosis or PT-NASH by liver biopsy, and four control recipients who had no signs of either condition on PT protocol biopsy. The average PT-BMI (range) across the groups were similar with controls at 31 (24-42), PT-steatosis at 29 (27-31), and PT-NASH at 31 (24-34). Other patient characteristics and histological findings are summarized in **Table 1**.

**Table 1:** Clinicopathological characteristics of transplant recipients included in the study.

Variable	Control (n=4)	Steatosis (n=2)	NASH (n=4)
Recipient age at transplant (y), mean (range)	55 (47-65)	46 (33-58)	54 (45-65)
Recipient sex (male), n (%)	1 (25)	2 (100)	3 (75)
Recipient weight (kg), mean (range)	81 (52-108)	81 (80-82)	86 (67-98)
Recipient height (cm), mean (range)	160 (148-169)	167 (160-173)	166 (160-170)
Recipient BMI (kg/m2), mean (range)	31 (24-42)	29 (27-31)	31 (24-34)
Donor age (y), mean (range)	33 (23-47)	56 (44-68)	31 (17-48)
Sex-matched donor, n (%) yes unknown	2 (50) 2 (50)	1 (50) 1 (50)	3 (75) 1 (25)
Donor type (deceased), n (%)	2 (50)	1 (50)	3 (75)
Primary indication for transplantation, n (%) NASH Alcohol HBV + HCC PSC Fulminant hepatitis HCV + Alcohol	3 (75) 1 (25) 0 (0) 0 (0) 0 (0) 0 (0)	0 (0) 0 (0) 1 (50) 1 (50) 0 (0) 0 (0)	2 (50) 0 (0) 0 (0) 0 (0) 1 (25) 1 (25)
Immunosuppressant, n (%) Tacrolimus Cyclosporine	4 (100) 0 (0)	2 (100) 0 (0)	3 (75) 1 (25)
Time from transplant to biopsy (y), mean (range)	2 (1-3)	5 (1-10)	5 (1-11)
Immunosuppressant serum levels (ng/mL), mean (range) Tacrolimus Cyclosporine	7.7 (6.1-9.5) N/A	7.3 (4.7-9.9) N/A	9.1 (7.7-9.8)^ns^ 349 (-)
AST (IU/L), mean (range)	18 (10-21)	48 (42-53)	48 (23-92)^ns^
ALT (IU/L), mean (range)	18 (8-20)	109 (72-146)	53 (33-97)*
ALP (IU/L), mean (range)	80 (67-96)	132 (108-155)	166 (67-415)^ns^
Dyslipidemia, n (%)	1 (25)	0 (0)	3 (75)
Diabetes Mellitus, n (%)	1 (25)	2 (100)	2 (50)
Hypertension, n (%)	3 (75)	1 (50)	3 (75)
Histology: Steatosis score, n (%) 0 (<5%) 1 (5-33%) 2 (>33-66%) 3 (>66%)	4 (100) 0 (0) 0 (0) 0 (0)	0 (0) 0 (0) 2 (100) 0 (0)	0 (0) 2 (50) 2 (50) 0 (0)
Histology: Lobular inflammation score, n (%) 0 (None) 1 (<2 foci/200x) 2 (2-4 foci/200x) 3 (>4 foci/200x)	4 (100) 0 (0) 0 (0) 0 (0)	1 (50 1 (50) 0 (0) 0 (0)	0 (0) 2 (500) 2 (50) 0 (0)
Histology: Ballooning score, n (%) 0 (None) 1 (Few balloon cells) 2 (Many cells/prominent ballooning)	4 (100) 0 (0) 0 (0)	0 (0) 0 (0) 0 (0)	0 (0) 3 (75) 1 (25)
Histology: NAFLD Activity Score (NAS), n (%) 0 (None) 1-2 (Mild) 3-4 (Moderate) 5-8 (Severe)	4 (100) 0 (0) 0 (0) 0 (0)	0 (0) 1 (50) 1 (50) 0 (0)	0 (0) 0 (0) 3 (75) 1 (25)
Histology: Fibrosis stage, n (%) F0 (absent) F1 (1 zone fibrosis) F2 (2 zone fibrosis) F3 (bridging fibrosis) F4 (cirrhosis)	4 (100) 0 (0) 0 (0) 0 (0) 0 (0)	2 (100) 0 (0) 0 (0) 0 (0) 0 (0)	2 (50) 1(25) 0 (0) 0 (0) 1(25)
More recent liver biopsy available, n (%)	0 (0)	2 (100)	2 (50)
Most recent histology: Fibrosis stage, n (%)† F0 (absent) F1 (1 zone fibrosis) F2 (2 zone fibrosis) F3 (bridging fibrosis) F4 (cirrhosis)	4 (100) 0 (0) 0 (0) 0 (0) 0 (0)	1 (50) 0 (0) 1 (50) 0 (0) 0 (0)	2 (50) 0 (0) 1 (25) 0 (0) 1 (25)

BMI, body mass index; NASH, non-alcoholic steatohepatitis; HCV, hepatitis C virus; HBV, hepatitis B virus; HCC, hepatocellular carcinoma; PSC, primary sclerosing cholangitis; Tx, transplantation; Bx, biopsy; AST, aspartate aminotransferase; ALT, alanine aminotransferase; ALP, alkaline phosphatase; dyslipidemia, low-density lipoprotein > 130 mg/dL (3.4 mmol/L), triglycerides > 150 mg/dL (1.7 mmol/L), or requirement of lipid-lowering agents; diabetes mellitus (DM): fasting plasma glucose (FPG) >=126 mg/dL (7.0 mmol/L) or 2-h plasma glucose>=200 mg/dL (11.1 mmol/L) during oral glucose tolerance test or A1C>=6.5% (48 mmol/mol) or requirement of anti-diabetic medications; hypertension: > 140/90 mm Hg, > 130/80 mm Hg with DM, or requirement of antihypertensive medications. †Most recent histology includes reports of liver biopsies performed after transcriptomic profiling was done. ns=not significant. *p-value of PT-NASH vs. control is 0.04

### 3.2 Comparison of gene expression in post-transplant and non-transplant NASH

**Figure 1:**
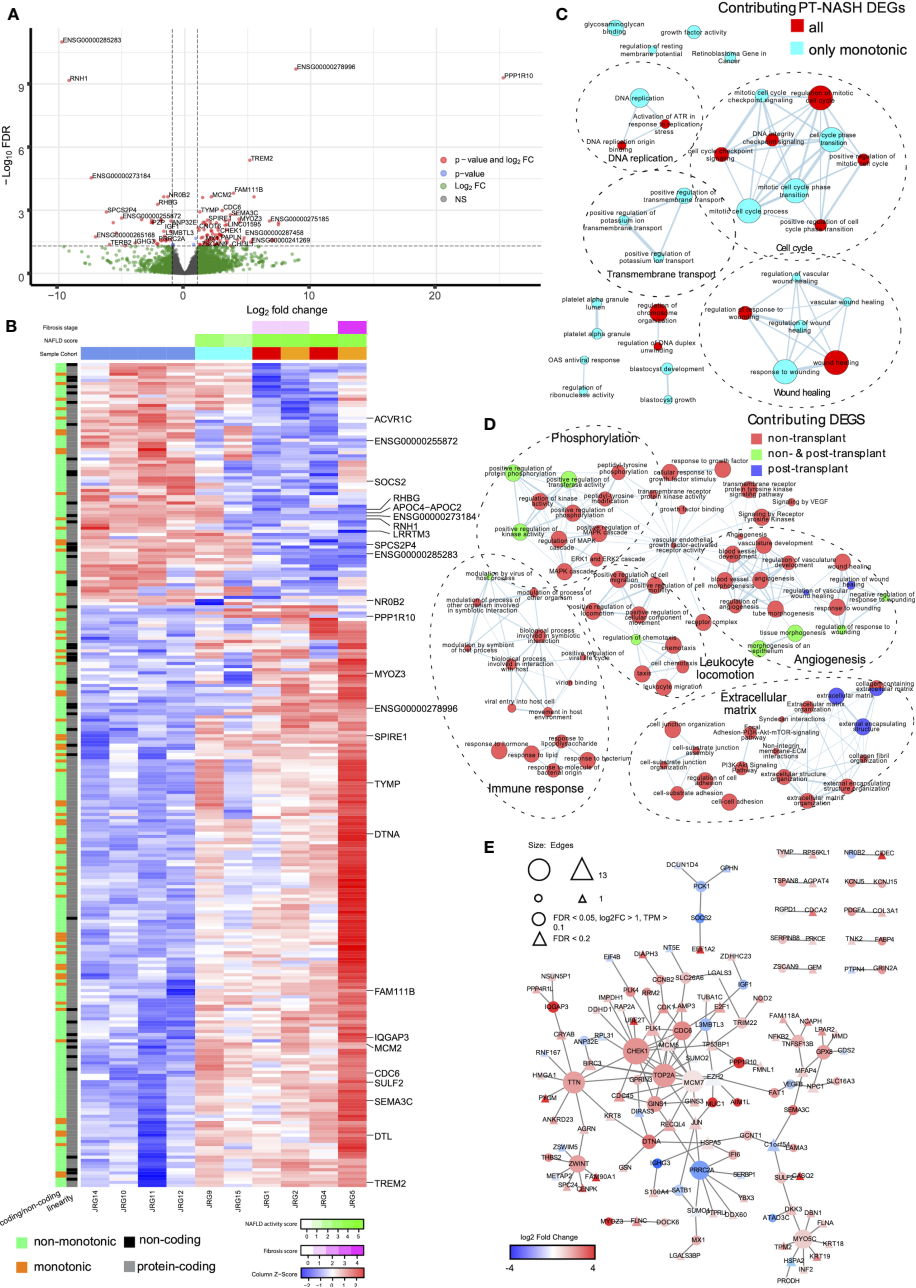
Gene expression patterns, enriched pathways, and protein-protein interaction (PPI) networks in livers with post-transplant and non-transplant NASH. **(A)** Volcano plot of differential gene expression in PT-NASH compared to control. Green: log2 FC ≥ 1; blue: FDR ≤ 0.05; red: log2 FC ≥ 1 and FDR ≤ 0.05. **(B)** Heatmap of TPM values of all PT-NASH DEGs. Sample annotations are shown at the top. The first row represents fibrosis score (purple), the second row represents NAFLD activity score (NAS, green); and the third row represents sample group (blue: control; cyan: steatosis; orange: *de novo* NASH; red: recurrent NASH). Gene annotations are shown on the left (orange: monotonic; green: non-monotonic; grey: protein-coding genes; black: lncRNAs). The 25 top DEGs are labelled on the right. **(C)** Enrichment map of pathways found in monotonic and non-monotonic PT-NASH expression patterns (ActivePathways, FDR < 0.2). Each pathway is a node, edges connect pathways sharing many genes, and colors indicate DEGs contribution to pathway enrichments (cyan: monotonic; red: combined monotonic and non-monotonic). Subnetworks were annotated manually to combine similar pathways (dashed circles). **(D)** Map of enriched pathways in PT-NASH and NT-NASH (ActivePathways, FDR < 0.2). Colors indicate disease type contributing to pathway enrichment (red: NT-NASH; blue: PT-NASH; green: combined). **(E)** PPI network interactions with DEGs in PT-NASH. Color indicates expression fold-change (PT-NASH *vs*. control livers), and circles show the most significant DEGs. Triangles indicate additional less-significant genes (FDR < 0.2).

We sequenced the transcriptomes of liver biopsies and identified 98 upregulated and 20 downregulated differentially expressed genes (DEGs) in PT-NASH samples compared to controls (**Figure 1A**, **Supplementary Table 1**). Principal component analysis (PCA) of the DEGs revealed that PT-NASH and control samples can be separated using the (PC2) = (PC1)/2 equation, while the principal component analysis with all genes placed all NASH samples linearly with the formula (PC2) = -(PC1)/6 ± 10000 (**Supplementary Figure 1**). Among the top 25 DEGs, protein phosphatase 1 regulatory subunit 10 (*PPP1R10*), IQ motif containing GTPase activating protein 3 (*IQGAP3*), and triggering receptor expressed on myeloid cells 2 (*TREM2*) were upregulated. Meanwhile, ribonuclease/angiogenin inhibitor 1 (*RNH1*) and nuclear receptor *NR0B2* were downregulated. We also identified two novel lncRNA genes: *ENSG00000278996* was exclusively expressed in PT-NASH while *ENSG00000273184* was only expressed in control.

To decipher the transcriptomic changes occurring throughout the disease course, we classified genes as having consistent increase or decrease in expression (i.e., monotonic) or no consistent change (i.e., non-monotonic) based on the changes in transcript abundance from control to PT-steatosis to PT-NASH states (**Figure 1B**, **Supplementary Figure 2**). Overall, 45% of genes (8,875) were classified as monotonic and 55% (10,972) as non-monotonic (**Supplementary Figure 3**). In contrast, the majority of significant DEGs (83% or 98/118) were monotonic, suggesting consistent increases or decreases of these differential expression patterns through disease evolution (**Supplementary Figure 3**).

To systematically compare our findings, we reanalyzed the previously published transcriptomes of NT-NASH with fibrosis (6) and identified 206 upregulated and 242 downregulated DEGs in NT-NASH (**Supplementary Table 2**). Interestingly, only 16% (19/118) of genes we identified as DEGs in PT-NASH were also found in NT, indicating differences in molecular disease phenotypes. The fractions of monotonic and non-monotonic gene expression patterns were similar in NT-NASH (41% monotonic), and most DEGs were classified as monotonic (61%, 274/448) (**Supplementary Figure 3**), confirming our observations in PT-NASH. Most DEGs shared between NT and PT were also monotonic (89%, 17/19).

### 3.3 Wound healing pathways are significantly dysregulated in post-transplant NASH

To identify dysregulated pathways in the disease samples, we performed joint functional enrichment analyses combining DEGs identified in our transcriptomics experiment (PT-NASH) and from an earlier study of NT-NASH (6). We identified collagen-containing ECM, extracellular organization, wound healing, and phosphoinositide-3-kinase-protein kinase B (PI3K-Akt) signaling pathways that were dysregulated regardless of transplantation status (**Table 2**, **Figure 1C**, **Supplementary Figures 4A, B**). Dysregulated pathways specific to PT-NASH included ECM (extracellular matrix, external encapsulating structure), vascular wound healing (regulation and response to wounding, angiogenesis, platelet alpha granule, glycosaminoglycan binding, heparin binding), and cell cycle (mitotic cell cycle, checkpoint signaling, DNA replication) (**Table 3**, **Figure 1D**, **Supplementary Figures 4C, D**). All significantly dysregulated pathways in PT-NASH and NT-NASH are provided in **Supplementary Tables 3**, **4**. To examine the common and distinct features of the identified pathways, we studied the shared DEGs found in the GO biological processes “collagen-containing extracellular matrix”, “extracellular structure organization”, “extracellular matrix organization”, “wound healing”, and “response to wounding” (**Supplementary Figure 5**). While the DEGs involved in “wound healing” and “response to wounding” were often shared (Jaccard coefficient 0.75), the DEGs involved in wound healing were mostly distinct from ECM-related genes (Jaccard coefficient 0.15). However, a few multifunctional genes were found in both types of processes, such as serpin family E member 1 (*SERPINE1)*, SPARC-related modular calcium binding protein 2 (*SMOC2)*, and collagen type III alpha 1 chain (*COL3A1)*.

**Table 2:** Significantly enriched pathways common to PT-NASH and NT-NASH.

Common dysregulated pathways in post-transplant and non-transplant NASH				
Term	Term ID	PT-NASH FDR	NT-NASH FDR	Top 5 Intersection Genes
GO:0062023	collagen-containing extracellular matrix	4.00E-03	3.50E-02	DCN, SEMA3B, SERPINB1, MYOC, TIMP2
GO:0043062	extracellular structure organization	2.70E-02	6.00E-03	CFLAR, ST7, ITGAL, ITGA3, ITGA2B
GO:0030198	extracellular matrix organization	2.70E-02	6.00E-03	CFLAR, ST7, ITGAL, ITGA3, ITGA2B
GO:0045229	external encapsulating structure organization	2.80E-02	6.00E-03	CFLAR, ST7, ITGAL, ITGA3, ITGA2B
GO:0042060	wound healing	2.80E-02	1.10E-02	ENPP4, CFLAR, TFPI, KDM1A, SLC4A1
GO:0009611	response to wounding	2.90E-02	1.10E-02	ENPP4, CFLAR, TFPI, KDM1A, SLC4A1
WP : WP4172	PI3K-Akt Signaling Pathway	4.70E-02	9.00E-03	BAD, ITGA3, ITGA2B, BRCA1, IGF1
WP : WP3932	Focal Adhesion-PI3K-Akt-mTOR signaling pathway	4.70E-02	9.00E-03	BAD, ITGA3, ITGA2B, BRCA1, IGF1

Pathways of post-transplant DEGs with 0.1 FDR and non-transplant DEGs with 0.01 FDR cut-offs are shown. Pathways were considered functionally related if at least 80% of DEGs in the pathways were common to both gene sets.

**Table 3:** Significantly enriched pathways specific to PT-NASH. Post-transplant DEGs with 0.1 FDR cut-offs are shown.

Dysregulated pathways specific to post-transplant NASH			
Term	Term ID	PT-NASH FDR	Top 5 Genes
GO:0061043	regulation of vascular wound healing	5.00E-05	ALOX5, SLC12A2, XBP1, SERPINE1, SMOC2
GO:0061042	vascular wound healing	4.60E-04	ADIPOR2, ALOX5, SLC12A2, MCAM, XBP1
GO:0005539	glycosaminoglycan binding	4.60E-04	CFH, AOC1, MPO, PAFAH1B1, PGLYRP1
GO:0001832	blastocyst growth	8.90E-04	IGF1, PALB2, GINS1, SALL4, NBN
GO:0060055	angiogenesis involved in wound healing	1.60E-03	CX3CL1, ADIPOR2, ALOX5, SLC12A2, MCAM
GO:0031012	extracellular matrix	1.85E-03	MMP25, ANOS1, DCN, SEMA3B, SERPINB1
GO:0061041	regulation of wound healing	1.85E-03	ENPP4, TFPI, ABCC8, TNFRSF12A, TBXA2R
GO:0030312	external encapsulating structure	1.85E-03	MMP25, ANOS1, DCN, SEMA3B, SERPINB1
GO:0031091	platelet alpha granule	7.13E-03	ITGA2B, CD9, IGF1, HGF, ACTN1
GO:1903034	regulation of response to wounding	7.13E-03	ENPP4, TFPI, ABCC8, TNFRSF12A, TBXA2R
GO:0008201	heparin binding	7.13E-03	CFH, AOC1, MPO, PAFAH1B1, TENM1
GO:1903047	mitotic cell cycle process	7.13E-03	MAD1L1, CDC27, PRKAR2B, KMT2E, FBXL3
GO:1905462	regulation of DNA duplex unwinding	7.30E-03	MNAT1, MCM2, SSBP1, POT1, TOP2A
GO:0007093	mitotic cell cycle checkpoint signaling	8.65E-03	MAD1L1, ZNF207, BRCA1, MRE11, SPDL1
GO:0001824	blastocyst development	9.27E-03	PSMC4, IGF1, CUL3, PTPN18, NLE1
GO:1901381	positive regulation of potassium ion transmembrane transport	9.50E-03	ABCC8, KCNQ1, KCNH2, WNK1, GAL
GO:0043268	positive regulation of potassium ion transport	1.48E-02	ABCC8, FHL1, KCNQ1, KCNH2, WNK1
GO:0006260	DNA replication	2.00E-02	RECQL, UPF1, LIG3, DBF4, PSMB1
GO:0030048	actin filament-based movement	2.00E-02	CACNA1G, CCDC88C, WAS, ATP1A2, VIM
GO:0061044	negative regulation of vascular wound healing	2.00E-02	ALOX5, SLC12A2, SERPINE1, MIR200B, TAFA5
GO:0000075	cell cycle checkpoint signaling	2.00E-02	MAD1L1, CRY1, ZNF207, BRCA1, MRE11
GO:0033044	regulation of chromosome organization	2.07E-02	MAD1L1, KDM1A, CDC27, UPF1, KMT2E
WP:2446	Retinoblastoma Gene in Cancer	2.07E-02	E2F2, ANLN, HLTF, SMC1A, MCM6
GO:0034764	positive regulation of transmembrane transport	2.07E-02	CFTR, CALCR, ABCC8, CACNG3, CX3CL1
GO:0044770	cell cycle phase transition	2.10E-02	MAD1L1, CDC27, UPF1, PRKAR2B, KMT2E
GO:0031093	platelet alpha granule lumen	2.10E-02	IGF1, HGF, ACTN1, ACTN2, TGFB2
GO:0045931	positive regulation of mitotic cell cycle	2.74E-02	POLDIP2, CDC27, KMT2E, PAFAH1B1, CUL3
GO:0051607	defense response to virus	2.74E-02	TSPAN6, MAP3K14, RNF216, POLR3B, ZMYND11
GO:0140546	defense response to symbiont	2.74E-02	TSPAN6, MAP3K14, RNF216, POLR3B, ZMYND11
GO:1901989	positive regulation of cell cycle phase transition	2.74E-02	CDC27, KMT2E, PAF1, CUL3, ANAPC4
REAC:R-HSA-176187	Activation of ATR in response to replication stress	2.74E-02	DBF4, RFC2, MCM10, MCM2, MCM6
GO:0001527	microfibril	2.74E-02	LTBP1, LTBP4, MFAP2, FBN2, MFAP1

Next, we analyzed the protein-protein interaction (PPI) networks of the DEGs identified in PT-NASH transcriptomes (**Figure 1E**). The network analysis highlighted mucin 1 (*MUC1*), absent in melanoma 1-like gene (*AIM1L*), and thrombospondin 2 (*THBS2*) among the upregulated genes, while the downregulated genes included insulin-growth factor 1 (*IGF1*), phosphoenolpyruvate carboxykinase 1 (*PCK1*), and suppressor of cytokine signaling 2 (*SOCS2*).

## 4 Discussion

In our comparison of PT-NASH to NT-NASH, we identified dysregulation of the PI3K-Akt signaling pathway, which has been associated with metabolic dysfunction, including obesity, metabolic syndrome, and NAFLD (16). Blocking PI3K signaling in hepatic stellate cells inhibits ECM deposition and reduces profibrotic factor expression (17). We also found processes of ECM organization to be affected regardless of transplantation status. Upregulated genes in PT-NASH included known biomarkers of NASH and advanced liver fibrosis, i.e., *MUC1*, *AIM1L* and *THBS2* (18–20). Among the downregulated genes were *IGF1*, a hormone-like insulin that is inversely associated with liver fibrosis in diabetes patients (21), *PCK1*, an activator of the PI3K pathway, whose downregulation causes aggravated fibrosis and inflammation in a NASH model (22), and *SOCS2*, a negative regulator of cytokine signaling that suppresses inflammation during NASH progression (23).

While our findings reiterate the molecular similarities of PT-NASH and NT-NASH, they also highlight the likely contribution of dysregulated tissue repair, i.e., wound healing and fibrosis, to the aggressive and faster progression of PT-NASH. Indeed, a comparison of our top 25 DEGs with an earlier meta-analysis of NT-NASH of 206 NAFLD patients (7) revealed greater overlap of wound healing or liver fibrosis and cirrhosis-related genes, including dystrobrevin alpha (*DTNA*) and sulfatase 2 (*SULF2*). Of note, none of our LT recipients received mTOR inhibitors as immunosuppressants, which are known to inhibit wound healing. Instead, these patients were treated with calcineurin inhibitors. Thus, the dysregulation of PI3K signaling and wound healing-related mechanisms we observed in PT-NASH were likely not a side effect of the immunosuppression therapy.

We found pathways related to wound healing to share numerous genes, and similar observation is true for ECM-related pathways. This is in part expected, as GO terms are structured in hierarchies, and those of the lower hierarchy will likely be identified separately despite belonging to a hierarchically higher pathway. Nonetheless, fewer genes were shared between pathways involved in wound healing and ECM processes, indicating that our pathway analysis of DEGs provides complementary findings.

The recent INTERLIVER study describes the molecular phenotypes of parenchymal injury of liver transplant biopsies and reports associations of steatohepatitis, fibrosis classifiers, and time since transplantation (24). Although the INTERLIVER cohort contained patients with other liver conditions coinciding with the development of *de novo* or recurrent NASH, such as recurrent hepatitis C, we were able to identify shared genes between our significant DEGs with their fibrosis classifier genes. These include members of the phosphoprotein phosphatase (*PPP*), family with sequence similarity (*FAM*), insulin-like growth factor (*IGF*), immunoglobulin heavy constant (*IGH*), solute carrier family (*SLC*), and sodium leak channel (NALCN) gene classes (24).

Most of the DEGs in PT-NASH that we identified are well-annotated protein-coding genes; however, two of the most significant results, ENSG00000285283 and ENSG00000278996, are novel lncRNA genes that have not been well described. A third novel lncRNA ENSG00000273184 was previously associated with angiogenesis and inflammation in major depressive disorder (25). miRNAs have been linked to NASH; therefore, as the differential expression of these lncRNAs correlates with disease status, they may serve as candidates for future PT-NASH experimental studies.

Transcriptomic data on NASH is scarce and publicly available datasets are often not well annotated clinically. This is further constrained in the case of liver transplantation. Although our study is limited by a low sample size, potential sampling biases, and disease heterogeneity, we believe our findings are of interest in the field of NASH. We profiled four cases of PT-NASH, including two recurrent and two *de novo* cases. Factors that contribute to the development of NASH are similar to those for PT-NASH (5, 26–28). Although the risk factors of recurrent and *de novo* PT-NASH are shared and include age, ethnicity, and the use of immunosuppressants, patients transplanted for NASH are at a higher risk of developing recurrence in the new liver graft. They are likely to have other metabolic syndromes such as obesity or diabetes mellitus, which are co-morbidities of NASH (29, 30). In fact, both our recurrent PT-NASH have high BMI (≥30) and a history of diabetes mellitus. On the other hand, donor-related factors including baseline steatosis and genetic makeup of the liver graft can contribute to both recurrent and *de novo* PT-NASH. Several genetic polymorphisms and miRNAs are associated with NT-NASH and the differential contribution of these factors originating from the donor or the recipient are currently of interest in studying PT-NASH (31, 32).

PT-NAFLD is a common complication attributed to the metabolic side effects of immunosuppressive medications, including weight gain and insulin resistance. A previous study reported a mean incidence of 57% and 40% for recurrent and *de novo* NAFLD at 3 years after LT, respectively (5). More importantly, PT-NAFLD and PT-NASH have accelerated fibrosis progression compared to NT patients with similar conditions (5). Our study, although limited with sample availability and variability, indicates that fibrosis may contribute to both the accelerated progression of PT-NASH. Organ fibrosis is a common debilitating process and previous studies have indicated the efficacy of fibrosis-targeting therapies in other organs (33, 34). Given the rapid acceleration of fibrosis in LT recipients diagnosed with PT-NASH, the use of fibrosis-targeting drugs to impede liver fibrosis and attenuate NASH development should be explored.

## Data availability statement

Raw data are available in ArrayExpress (accession E-MTAB-11688).

## Ethics statement

The studies involving human participants were reviewed and approved by University Health Network Research Ethics Board. The ethics committee waived the requirement of written informed consent for participation.

## Author contributions

MB and JR conceptualized the research design. AA identified eligible patient samples. EP contributed to sample acquisition. CB contributed to data analysis. SF performed histological assessment. DP and KP performed the research and data analysis. KP, DP, MB, and JR wrote the manuscript. All authors contributed to the article and approved the submitted version.

## Supplementary material

Supplementary Table 1 Significant DEGs identified in PT-NASH compared to post-transplant control.

Supplementary Table 2 Significant DEGs identified in NT-NASH compared to non-transplant control.

Supplementary Table 3 Significantly dysregulated pathways specific to PT-NASH.

Supplementary Table 4 Significantly dysregulated pathways specific to NT-NASH.

## Glossary

**Table:**

AIM1L	absent in melanoma 1-like
BMI	body mass index
COL3A1	collagen type III alpha 1 chain
DEG	differentially expressed genes
DTNA	dystrobrevin alpha
ECM	extracellular matrix
FAM	family with sequence similarity
FC	fold change
FDR	false discovery rate (i.e., adjusted p-value)
GSE	Gene Expression Omnibus Series
GO	Gene Ontology
HCC	hepatocellular carcinoma
IGF1	insulin-growth factor 1
IGH	immunoglobulin heavy constant
IQGA	IQ motif containing GTPase activating protein 3
lncRNA	long non-coding ribonucleic acid
LT	liver transplantation
MUC1	mucin 1
NAFLD	non-alcoholic fatty liver disease
NALCN	sodium leak channel
NAS	NAFLD activity scoring
NASH	non-alcoholic steatohepatitis
NR0B2	nuclear receptor subfamily 0 group B member 2
NT	non-transplant
OICR	Ontario Institute for Cancer Research
PCK1	phosphoenolpyruvate carboxykinase 1
PI3K-Akt	phosphoinositide-3-kinase-protein kinase B
PPI	protein-protein interaction
PPP	phosphoprotein phosphotase
PPP1R10	protein phosphatase 1 regulatory subunit 10
PT	post-transplant
REB	Research Ethics Board
RNH1	ribonuclease/angiogenin inhibitor 1
SERPINE1	serpin family E member 1
SLC	solute carrier family
SMOC2	SPARC-related modular calcium binding protein 2
SOCS2	suppressor of cytokine signaling 2
SULF2	sulfatase 2
THBS2	thrombospondin 2
TPM	transcript per million
TREM2	triggering receptor expressed on myeloid cells 2
UHN	University Health Network
