# Supplementary material for: Transcriptomic changes in liver transplant recipients with non-alcoholic steatohepatitis indicate dysregulation of wound healing
Source: Front Endocrinol (Lausanne). 2023 May 8;14:1111614. doi: 10.3389/fendo.2023.1111614 (PMC10200958; doi:10.3389/fendo.2023.1111614)
Supplement: Supplementary file 2 [file DataSheet_1.docx]

Supplementary Material

**Transcriptomic Changes in Liver Transplant Recipients with Non-Alcoholic Steatohepatitis Indicate Dysregulation of Wound Healing**

**Diogo Pellegrina^1^†, Khairunnadiya Prayitno^2^†, Amirhossein Azhie^2^, Elisa Pasini^2^, Cristina Baciu^2^, Sandra Fischer^3^, Jüri Reimand^1,4,5^*, Mamatha Bhat^2,6,7^***

**†**These authors contributed equally to this work and share first authorship

*****These authors contributed equally to this work and share last authorship

***Correspondence:**Mamatha Bhat
Mamatha.Bhat@uhn.ca

Jüri Reimand
Juri.Reimand@utoronto.ca

# Supplementary Figures


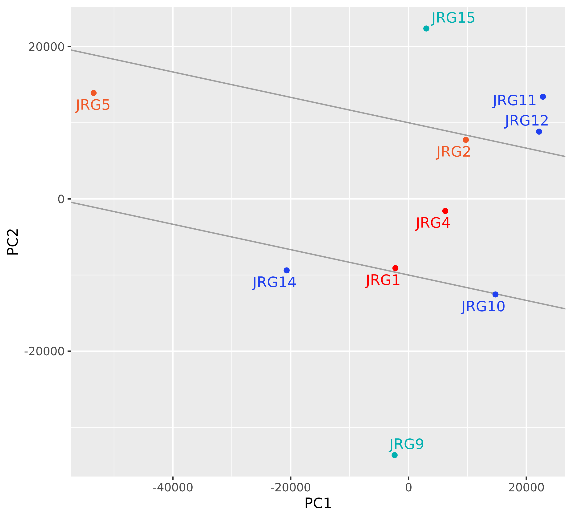

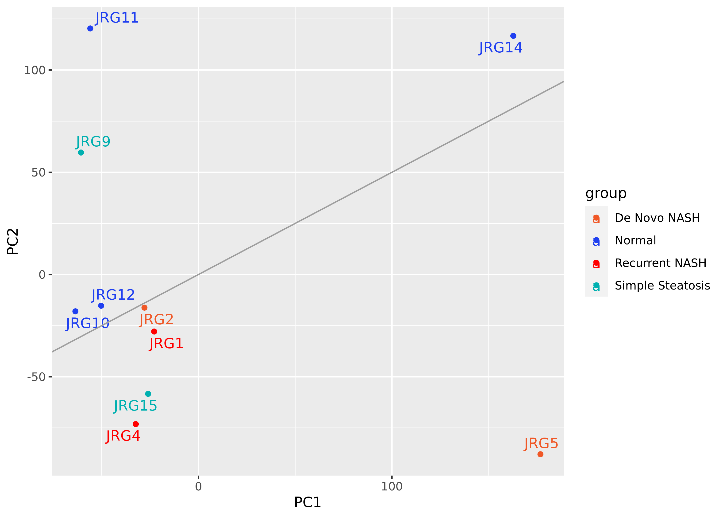


**Supplementary Figure 1. A)** Principal component analysis (PCA) of A) all observed genes and B) DEGs in PT-NASH. Grey lines separate PT-NASH and control samples.

**Supplementary Figure 2.** Gene expression dynamics of all measured genes in the post-transplant control, steatosis, and NASH samples. Monotonic genes (left) displayed continuous, one-directional expression change, either increasing (top) or decreasing (bottom) through disease states. Non-monotonic genes showed either a minimum (middle panel) or a maximum (right panel) expression in steatosis, such that expression was either higher (top panel) or lower (bottom panel) in NASH compared to control.

**Supplementary Figure 3.** Fractions of all genes and significant DEGs in post-transplant and non-transplant conditions that were monotonic (blue) or non-monotonic (red). Numbers indicate the total genes in each group.

**Supplementary Figure 4.** Comparison of post-transplant and non-transplant gene expression. Gene expression changes belonging to A) collagen-containing extracellular matrix, B) PI3K-Akt signaling, C) regulation of wound healing, and D) cell cycle checkpoint pathways between control and NASH in post-transplant and non-transplant conditions.

**Supplementary Figure 5.** UpSet plot of shared DEGs (PT-NASH or NT-NASH) for “collagen-containing extracellular matrix”, “extracellular structure organization”, “extracellular matrix organization”, “wound healing”, and “response to wounding” pathways.
